# MicroRNA-7 sensitizes non-small cell lung cancer cells to paclitaxel

**DOI:** 10.3892/ol.2014.2500

**Published:** 2014-09-04

**Authors:** RONGHUA LIU, XIAOMING LIU, YIJIE ZHENG, JIE GU, SHUDAO XIONG, PEI JIANG, XUECHAO JIANG, ENYU HUANG, YIXIAN YANG, DI GE, YIWEI CHU

**Affiliations:** 1 Department of Immunology, Shanghai Medical College, Key Laboratory of Molecular Medicine of Ministry of Education, Fudan University, Shanghai 200032, P.R. China; 2 Biotherapy Research Center of Fudan University, Shanghai 200032, P.R. China; 3 Department of Thoracic Surgery, The Affiliated Zhongshan Hospital of Fudan University, Shanghai 200032, P.R. China; 4 Department of Hematology/Oncology, The Second Hospital of Anhui Medical University, Hefei, Anhui 230601, P.R. China

**Keywords:** microRNA, chemotherapy, drug resistance, lung cancer, EGFR

## Abstract

Paclitaxel (PTX) is the front-line chemotherapeutic agent against human non-small cell lung cancer (NSCLC). However, its therapeutic efficacy is restricted by the increasing frequency of chemotherapeutic resistance in NSCLC. Accumulating evidence has shown the potential role of microRNAs (miRNAs) in the chemotherapeutic sensitivity of cancer cells. Previously it was reported that microRNA-7 (miR-7) acts as an important tumor suppressor in NSCLC. Therefore, the present study was conducted to determine the regulatory role of miR-7 in PTX chemotherapy for NSCLC. Four NSCLC cell lines were used to analyze the correlation of the PTX-sensitivity and endogenoaus miR-7 expression. miR-7 expression was up- and downregulated using miR-7 mimics and inhibitors respectively, and the role of miR-7 in sensitizing NSCLC cells to PTX was assessed by cell viability and apoptosis assays. The molecular mechanism of PTX sensitivity was determined by quantitative polymerase chain reaction and western blotting. It was found that the sensitivity of NSCLC cells to PTX was dependent on endogenous miR-7. Upregulation of miR-7 enhanced the PTX-sensitivity of NSCLC cells by suppressing cell proliferation and promoting cell apoptosis, while the inhibition of miR-7 abrogated the antiproliferative proapoptotic effects of PTX. Pretreatment of miR-7 mimics enhanced the PTX-mediated downregulation of epidermal growth factor receptor (EGFR) in NSCLC cells. These results have identified miR-7 as a potential EGFR-targeting sensitizer in PTX therapy. These data may facilitate the development of novel chemotherapeutic approaches for NSCLC.

## Introduction

Non small-cell lung cancer (NSCLC) is one of the most commonly diagnosed types of cancer and a leading cause of mortality worldwide (1). Chemotherapy is a crucial strategy for advanced-stage NSCLC, and paclitaxel (PTX) is employed as a front-line chemotherapeutic agent in clinical oncology (2,3).

PTX is a member of the taxanes family that promotes microtubule assembly and interferes with signal transduction (4,5). The potent antitumor efficacy of PTX is mediated by the direct induction of DNA damage and cell death by apoptosis (4,6,7). However, the efficiency of PTX-based cancer chemotherapy is increasingly limited by the development of therapeutic resistance (8–10). Thus, the challenge for improving chemotherapeutic efficacy is in the development of strategies that enhance cancer cell sensitivity to treatment.

MicroRNAs (miRNAs) are small, non-coding RNAs of ~22 nucleotides. Mature miRNAs partially bind to their target mRNAs at complementary sites in the 3′-untranslated region (3′-UTR), and regulate gene expression (11,12). miRNAs are responsible for various biological and pathological processes, including cancer development and progression (11–14). Recent studies have reported several miRNAs that enhance chemotherapeutic efficacy by modulating the sensitivity of cancer cells to conventional chemotherapeutic drugs (15,16). It has been shown that miR-221/222 confers tamoxifen resistance in breast cancer (17); miR-137 sensitizes doxorubicin-resistant neuroblastoma cells to doxorubicin, as shown by reduced proliferation and increased apoptosis (18); and miR-21 confers cisplatin resistance in gastric cancer cells (19). Other miRNAs, including miR-328 (20), miR-326 (21), and miR-34a (22), have also been shown to modulate chemosensitivity.

We previously reported miR-7 as a key tumor suppressor in NSCLC that could suppress cell proliferation, induce apoptosis, inhibit cancer migration and reduce tumorigenicity in A549 adenocarcinomic human alveolar basal cells (23). The function of miR-7 in PTX-mediated chemotherapy for NSCLC, however, has not been investigated. Given that our previous study demonstrated the importance of miR-7 in NSCLC pathogenesis, the present study hypothesized that miR-7 plays a crucial role in regulating NSCLC sensitivity to PTX.

To the best of our knowledge, this study provides the first evidence of the potential utility of miR-7 as a sensitizer in PTX therapy for NSCLC and thus provides a novel molecular target for therapeutic development.

## Materials and methods

### Tissue samples and cell lines

Human NSCLC and matched adjacent tissues were obtained from 20 patients at Zhongshan Hospital, Fudan University (Shanghai, China) between 2008 and 2011. None of these patients had received chemotherapy prior to surgery. This study was carried out according to the World Medical Association Declaration of Helsinki and was approved by the Medical Ethics Committee of Zhongshan Hospital.

A549, H1395 human adenocarcinoma lymphoblasts, 95C and 95D low and high metastatic human lung cancer cells, were obtained from the Institute of Biochemistry and Cell Biology of the Chinese Academy of Science (Shanghai, China). The HBE human bronchial epithelial cell line was obtained from Xiangfu Bio (Shanghai, China). These cell lines originated from the American Type Culture Collection Manassas, VA, USA. A549, H1395, and HBE were grown in Dulbecco’s modified Eagle’s medium (DMEM) supplemented with 10% fetal bovine serum (FBS), 2 μM glutamine, 100 IU/ml penicillin, and 100 μg/ml streptomycin sulfate. 95C and 95D cells were cultured in RPMI-1640 media, supplemented with 10% FBS, 2 μM glutamine, 100 IU/ml penicillin, and 100 μg/ml streptomycin sulfate.

### Quantitative polymerase chain reaction (qPCR) for miRNA

Total RNA was extracted using TRIzol^®^ reagent (Invitrogen Life Sciences, Carlsbad, CA, USA) and reverse-transcribed with miR-7-specific primers (Guangzhou RiboBio Co., Ltd., Guangzhou, China). qPCR was performed using an ABI 7500 thermocycler (Applied Biosystems, Foster City, CA, USA) starting with 1 μl cDNA and SYBR^®^-Green Realtime PCR Master Mix (Toyobo Co., Ltd., Osaka, Japan). The relative amount of each miRNA was normalized to the amount of U6. The primer sequences for U6 and miR-7 are listed in Table I.

### miRNA transfection

The human miR-7 mimics (miR-7) (dsRNA oligonucleotides), miR-7 inhibitor (single-stranded chemically modified oligonucleotides), negative control mimic (miR-NC), and negative control inhibitor (Ctrl inhibitor) were purchased from RiboBio (Guangzhou RiboBio Co., Ltd). Cells were seeded in 24-well plates (5×10^4^ cells/well) for 24 h. When the cells were 30–50% confluent, they were transfected with miR-7 or miR-7 inhibitor (50 nmol final concentration) using Lipofectamine^®^ 2000 (Invitrogen Life Technologies). Six hours after transfection, the cells were maintained in DMEM with 10% FBS for all subsequent treatments.

### qPCR for EGFR

Following transfection with miR-7 or miR-NC for 24 h, A549/H1299 cells were treated with PTX (0.1 μM) for 24 h. qPCR was performed in triplicate for each sample using the PrimerScript RT reagent kit (Takara Bio, Inc., Dalian, China). GAPDH was used for normalization. Primers for GAPDH and EGFR were designed using Primer Express^®^ (Invitrogen Life Technologies) and are listed in Table I.

### Western blotting

Proteins were extracted from A549 or 95D cells following transfection with miR-7, miR-NC, miR-7 inhibitor, or Ctrl inhibitor for 48 h using SDS lysis buffer (P0013G, Beyotime, Shanghai China). Primary antibodies included anti-EGFR (1:2,000) and anti-GAPDH (1:10,000) (Cell Signaling, Danvers, MA, USA). The procedure for western blotting was performed as described previously (14). Equal amounts of protein were transferred onto polyvinylidene difluoride membranes (Merck Millipore, Darmstadt, Germany)following resolution by SDS-PAGE (P0012A, Beyotime). The membranes were blocked in 5% non-fat milk and probed with the primary monoclonal rabbit anti-human anti-EGFR (1:2,000) and monoclonal mouse anti-human anti-GAPDH (1:10,000) antibodies (Cell Signaling). The membranes were then washed and incubated with a polyclonal goat anti-mouse or polyclonal goat anti-rabbit secondary antibody conjugated to horseradish peroxidase. The specific bands were detected by enhanced chemiluminescence (34095, Thermo Fisher Scientific, Waltham, MA, USA)

### Cell proliferation assay

Following transfection for 48 h, the cell proliferation was measured using a Cell Counting Kit-8 assay (CCK-8; Dojindo, Kunamoto, Japan), based upon a redox assay similar to the MTT assay.

To analyze the effects of miRNA in combination with PTX, the cells transfected with miR-7 or miR-NC were treated with PTX at concentrations of 0, 10, 20, 40, 80 and 160 nM for 48 h. The cell proliferation assays were performed using the CCK-8 assay according to the manufacturer’s instructions.

The sensitivity of each type of cell to PTX was evaluated using CCK-8, presented as the cell viability (%). The concentration of PTX at which 50% of cells survived presents the IC_50_ value.

### Apoptosis

A549 or 95D cells were seeded in 24-well plates, incubated for 24 h, and then transfected with miR-7, miR-NC, miR-7 inhibitor, or Ctrl inhibitor for a further 24 h. Following transfection, the cells were treated with PTX (20 nM) for 24 h and then collected. The level of apoptosis was analyzed by flow cytometry and Annexin V/propidium iodide (PI) staining.

### Statistical analysis

Data are presented as the mean ± standard error of the mean, from replicate experiments (n>3). The results were analyzed using PRISM 5.0 (GraphPad Software Inc., San Diego, CA, USA). A Student’s t-test was used to analyze intergroup differences for two groups, and analysis of variance was used to analyze >2 groups. P<0.05 was considered to indicate a statistically significant difference.

## Results

### PTX-sensitivity of NSCLC cells is dependent on endogenous miR-7 expression

**Figure 1:**
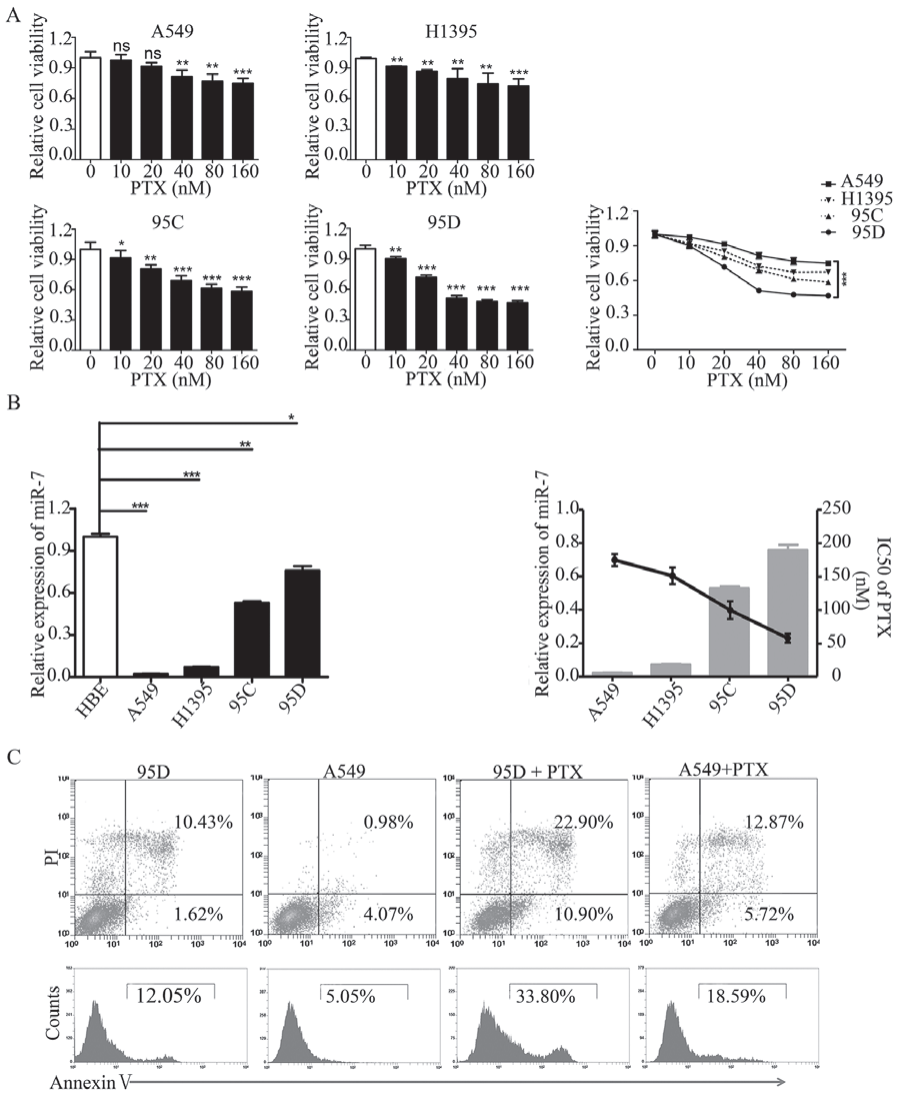
PTX sensitivity of non-small cell lung cancer (NSCLC) cells is positively correlated with endogenous miR-7 expression. (A) Cells were treated with different doses of PTX (0, 10, 20, 40, 80 and 160 nM) for 48 h. The cell viability was determined using a Cell Counting kit-8 assay (^***^P<0.001). (B) MiR-7 expression in NSCLC cell lines was measured by quantitative polymerase chain reaction (^*^P<0.05, ^**^P<0.01, ^***^P<0.001). The sensitivity to PTX of A549, H1395, 95C and 95D cells was based on their IC_50_ (207.8±1.38, 159.6±1.42, 131.3±0.74 and 87.94±1.41 nM, respectively). (C) Apoptosis of A549 and 95D cells was detected by Annexin V/PI assay following 24-h exposure to PTX. Results are representative of 2–3 independent experiments. Error bars are the standard error of the mean. PTX, paclitaxel; A549, adenocarcinomic human alveolar basal epithelial cells; H1395, human adenocarcinoma lymphoblasts; 95C, low metastatic human lung cancer cells; 95D, high metastatic human lung cancer cells; PI, propidium iodide; IC_50_, half maximal inhibitory concentration; miR-7, microRNA-7.

PTX sensitivity was detected in various NSCLC cell lines (A549, H1395, 95C, and 95D) of differing origins by *in vitro* cell viability assays. Cell viability was observed to be reduced by PTX in a dose-dependent manner (Fig. 1A). Although the trends were similar, the sensitivity of the four NSCLC cell lines to PTX differed [half maximal inhibitory concentration (IC_50_) A549: 207.8±1.38 nM; H1366: 159.6±1.42 nM; 95C: 131.3±0.74 nM; 95D: 87.94±1.41 nM), suggesting that other factors may mediate PTX sensitivity. Our previous screen detected miR-7 expression in these cell lines (24). In the present study, miR-7 expression was shown to be frequently downregulated in these NSCLC cell lines as compared with the normal lung epithelial cell line (HBE). It was also found that miR-7 expression varied in different cell lines, similar to the variation observed in PTX sensitivity. It was observed that a higher expression of miR-7 in 95D cells correlated with a higher sensitivity of this cell line to PTX, strongly suggesting a positive association between PTX sensitivity and endogenous miR-7 expression (Fig. 1B). Furthermore, 95D cells showed a higher apoptotic frequency as compared with A549 cells following exposure to PTX (10.90 vs. 5.72% in early apoptosis, 22.90 vs. 12.87% in later apoptosis) (Fig. 1C). These findings indicated that PTX sensitivity in NSCLC cell lines is dependent on endogenous miR-7 expression.

### Overexpression of miR-7 sensitizes NSCLC cells to PTX

**Figure 2:**
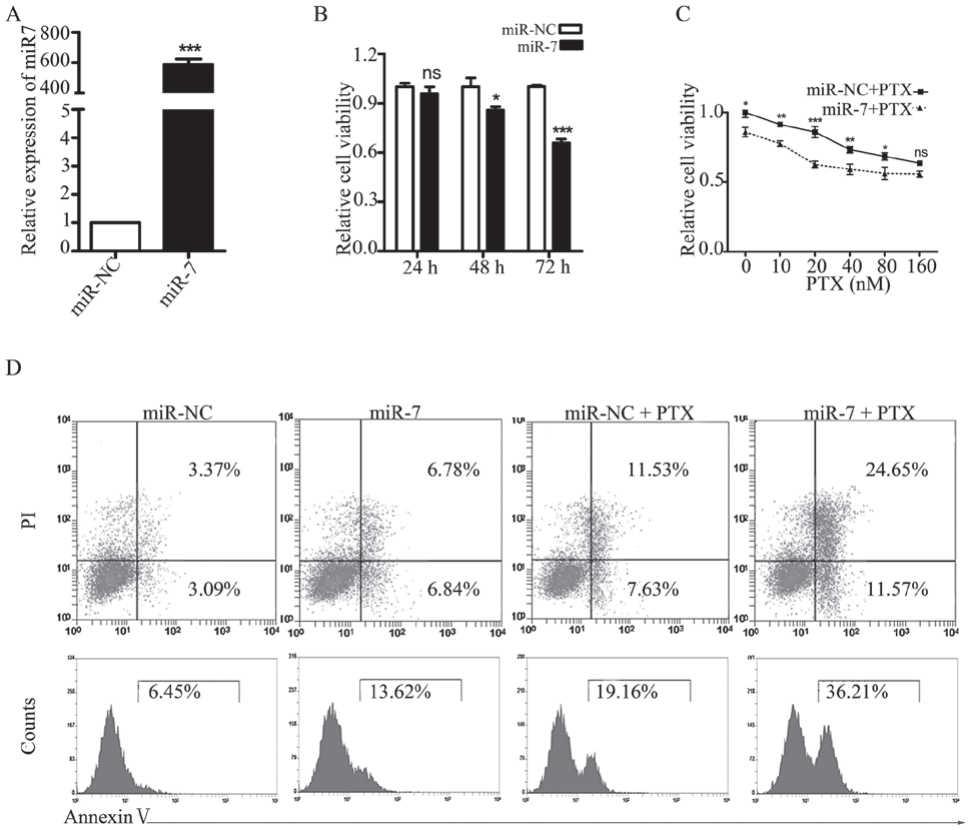
Overexpression of miR-7 in A549 adenocarcinomic human alveolar basal epithelial cells, increases cellular sensitivity to PTX. (A) MiR-7 expression in A549 cells, following transfection with miR-7 or miR-NC, was determined by quantitative polymerase chain reaction (^***^P<0.001). (B) Cell viability of A549 cells was performed by a Cell Counting kit-8 (CCK-8) assay following transfection with miR-7 mimics or miR-NC (^*^P<0.05, ^***^P<0.001). (C and D) A549 cells transfected with miR-7 mimics or miR-NC were treated with PTX for 48 h. Cell viability and apoptosis were evaluated by the CCK-8 and Annexin V/PI assays (^*^P<0.05, ^**^P<0.01, ^***^P<0.001). Results are representative of 2–3 independent experiments. Error bars are the standard error of the mean. PTX, paclitaxel; miR-7, microRNA-7; miR-NC, microRNA-negative control mimic; PI, propidium iodide.

It was hypothesized that the upregulation of miR-7 increases the sensitivity of NSCLC cells to PTX. A549 cells, a line with lower miR-7 expression, was selected as an *in vitro* model. miR-7 was overexpressed in A549 cells by transfection with miR-7 mimics, which resulted in the inhibition of A549 cell proliferation, especially after 72 h (Fig. 2A and B). To determine whether the increased expression of miR-7 would enhance sensitivity to PTX, cells transfected with miR-7 or miR-NC mimics were treated with PTX and viability was measured using the CCK-8 assay. Pre-treatment with miR-7 mimics enhanced PTX-mediated suppression of A549 cell viability, most notably at lower doses of PTX. It was observed that 20 nM PTX combined with miR-7 mimics was as effective as 80 nM PTX treatment alone (Fig. 2C). These data showed that the overexpression of miR-7 enabled reduction of the dose of PTX used for treatment.

Apoptosis is the predominant mechanism of PTX-induced toxicity. An Annexin V/PI assay was therefore used to measure the apoptotic frequency in miR-7-transfected A549 cells treated with PTX (20 nM). In comparison to PTX treatment alone, PTX and miR-7 overexpression induced apoptosis in A549 cells (11.57 vs. 7.63% in early apoptosis and 24.65 vs. 11.53% in later apoptosis) (Fig. 2D). Therefore, miR-7 expression sensitized the cells to PTX-induced apoptosis, thus enhancing its cytotoxic effect.

### Inhibition of miR-7 promotes NSCLC cell resistance to PTX

**Figure 3:**
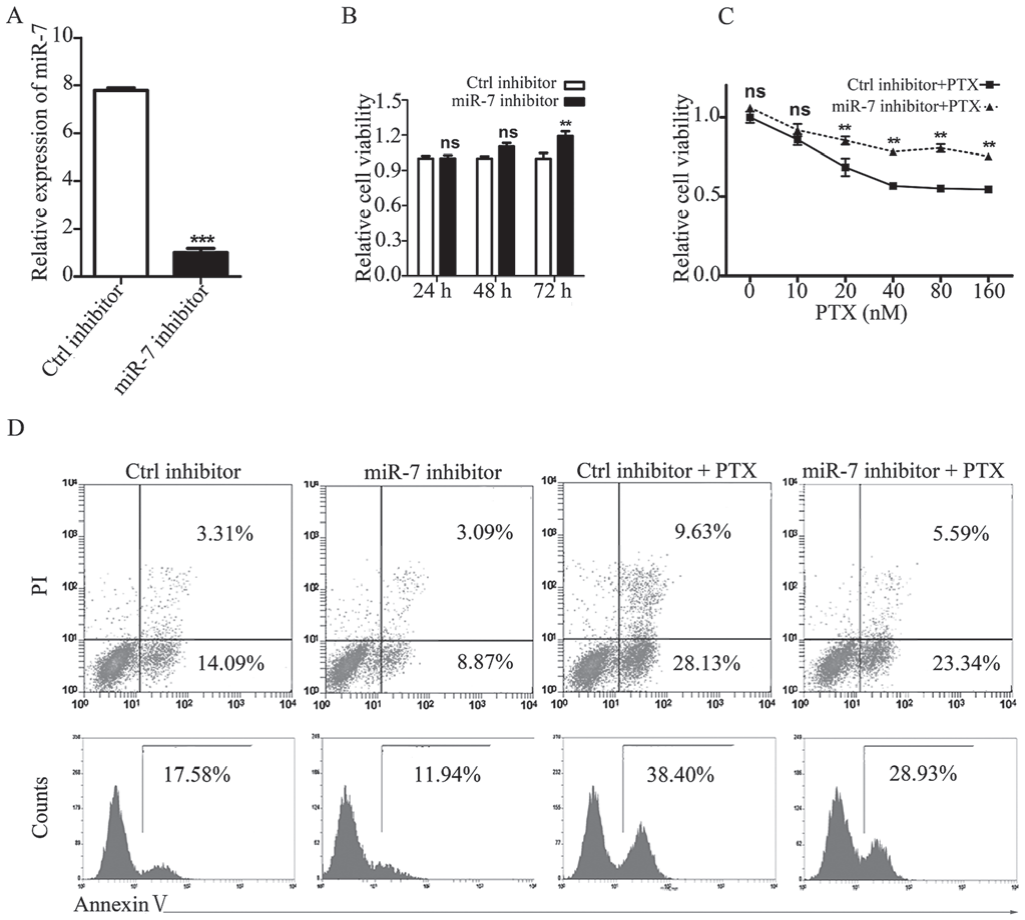
Knockdown of endogenous miR-7 expression in 95D high metastatic human lung cancer cells enhances cell resistance to PTX. (A) MiR-7 expression in 95D cells following transfection with the miR-7 or Ctrl inhibitor was detected by quantitative polymerase chain reaction (^***^P<0.001). (B) Cell viability of 95D cells was evaluated by Cell Counting kit-8 (CCK-8) assay following transfection with miR-7 or Ctrl inhibitor (^**^P<0.01). (C and D) Viability and apoptosis of 95D cells was determined by the CCK-8 and Annexin V/PI assays 48 h after transfection with the miR-7 inhibitor and PTX combined treatment (^*^P<0.05, ^**^P<0.01). Results are representative of 2–3 independent experiments. Error bars are the standard error of the mean. PTX, paclitaxel; PI, propidium iodide; ns, non-significant; miR-7, microRNA-7; Ctrl inhibitor, negative control inhibitor.

To investigate the role of miR-7 in PTX sensitivity, the high miR-7-expressing PTX-sensitive NSCLC cell line, 95D, was used as an *in vitro* model. MiR-7 inhibitor was used to knock down endogenous miR-7 (Fig. 3A). Following 72-h knockdown, the proliferation of 95D cells was observed to be increased (Fig. 3B).

Following transfection with the miR-7 or control inhibitor, 95D cells were treated with PTX at various concentrations. As expected, the combined treatment of 95D cells with an miR-7 inhibitor and PTX restored the PTX-mediated suppression of cell viability and apoptosis (Fig. 3C and D). Therefore, both the gain- and loss-of-function experiments indicated an important function for miR-7 in the PTX sensitivity of NSCLC cells.

### miR-7-enhanced PTX sensitivity in NSCLC cells is mediated by EGFR

**Figure 4:**
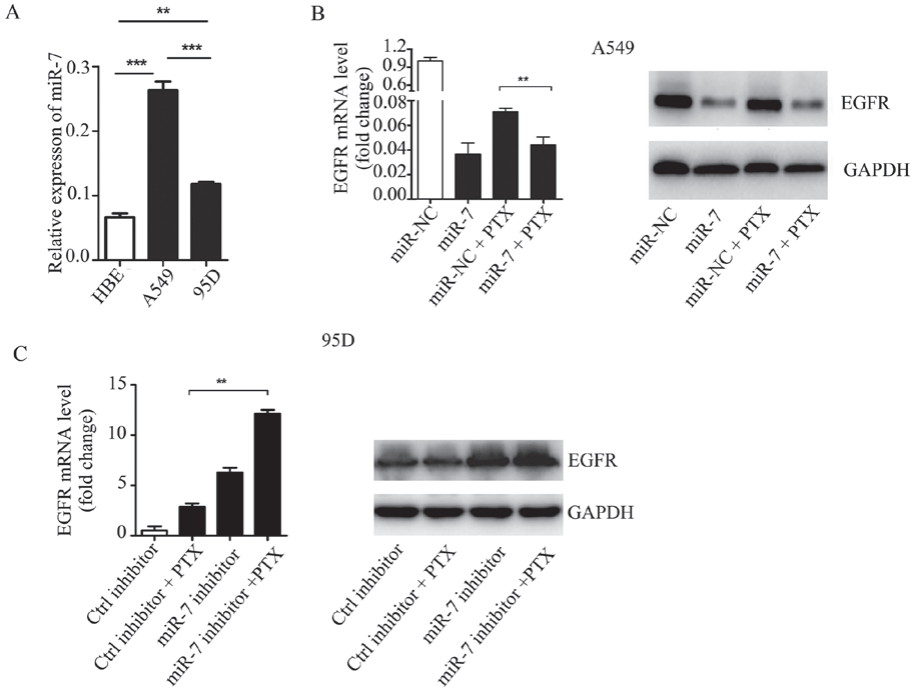
EGFR is involved in the enhancement of miR-7-mediated PTX sensitivity. (A) EGFR expression in A549 and 95D cells was detected using quantitative polymerase chain reaction (qPCR) (^**^P<0.01, ^***^P<0.001). (B) EGFR mRNA level was measured with qPCR (^**^P<0.01). EGFR protein level was examined by western blotting using GAPDH as a loading control. (C) EGFR mRNA and protein levels were examined by qPCR and western blotting (^**^P<0.01). Error bars are the standard error of the mean. PTX, paclitaxel; miR-7, microRNA-7; Ctrl inhibitor, negative control inhibitor; HBE, human bronchial epithelial cells; miR-NC, microRNA-negative control mimic; 95D, high metastatic human lung cancer cells; A549, adenocarcinomic human alveolar basal epithelial cells; EGFR, epidermal growth factor receptor.

Given the function of EGFR in NSCLC development and apoptosis, it was hypothesized that EGFR is involved in miR-7-mediated NSCLC cell sensitivity to PTX. qPCR showed that EGFR was upregulated in NSCLC A549 and 95D cells as compared with HBE cells (Fig. 4A). Furthermore, EGFR expression was significantly higher in A549 cells as compared with 95D cells. An inverse correlation was observed between the endogenous miR-7 expression levels and PTX sensitivities.

The expression of EGFR was then examined in A549 cells treated with miR-7 mimics, PTX, or both, by qPCR and western blotting. Overexpression of miR-7 inhibited EGFR expression in A549 cells. In comparison to PTX plus miR-NC, the cells treated with PTX following pretreatment with miR-7 mimics exhibited a significantly lower EGFR expression, at the mRNA and protein level (Fig. 4B). Conversely, the inhibition of miR-7 was associated with an increased mRNA and protein expression of EGFR in 95D cells treated with PTX (Fig. 4C). These data indicate that EGFR may mediate the molecular mechanism of NSCLC sensitization to PTX by miR-7.

## Discussion

Accumulating reports have indicated that chemotherapeutic treatments alter miRNA profiles in cancer (15,25,26). Several microRNAs, including miR-203b (27), miR-21 (28), and miR-137 (18) may also contribute to chemotherapeutic efficacy. In the present study, miR-7 was identified as an important molecule in PTX treatment. It was first shown that PTX sensitivity is dependent on endogenous miR-7 expression in NSCLC cell lines. Overexpression of miR-7 sensitized NSCLC cells to PTX, mainly by promoting PTX-induced cell apoptosis. Furthermore, administration of lower doses of PTX following pretreatment with miR-7 mimics, produced similar antitumor effects as compared with higher doses of PTX treatment alone (Fig. 2C). This finding suggests that miR-7 may allow for a reduction in the PTX doses used in chemotherapy and help address the problem of therapeutic resistance in NSCLC.

MiR-7 is a putative tumor suppressor in a large range of solid tumors, and is often downregulated in NSCLC (29). Results of the present study have shown that the expression of miR-7 varied in different NSCLC cell lines, since these cell lines originated from different individuals. The interindividual variability of miR-7 expression was confirmed (data not shown). In NSCLC, miR-7 has been reported to suppress tumorigenesis by targeting a number of important proto-oncogenes, including EGFR, IRS1 (insulin receptor substrate 1), IRS2, RAF1 (v-raf-1 murine leukemia viral oncogene homologue 1), and PAK1 (p21/CDC42/RAC1-activated kinase 1), and by inhibiting EGFR/AKT pathway activation (30–33). Additionally, inhibition of cell proliferation is associated with EGFR downregulation following transfection of miR-7 into NSCLC cells. Combined treatment with PTX and miR-7 mimics reduced EGFR expression at the mRNA and protein levels. By contrast, the miR-7 inhibitor increased EGFR expression in PTX-treated cells. These data indicate that miR-7-mediated enhancement of A549 chemosensitivity to PTX may occur through EGFR targeting.

The present study has focused on EGFR since it was previously identified as a critical target of miR-7 in many solid tumors, including NSCLC, and it contributes to tumor progression and poor prognosis (30,32,34). EGFR also functions in chemotherapeutic resistance and radiation tolerance in tumor cells, in which multiple downstream pathways of EGFR, including RAS-RAF-MAK-MAKP, PI3K/AKT, and STAT, are activated (31,33,35–38). Lee *et al* (38) reported that the overexpression of miR-7 increases the radiosensitivity of various human cancers by directly suppressing the activation of EGFR-PI3K-AKT. The present study has demonstrated that EGFR functions in miR-7-enhanced chemosensitivity to PTX, and a similar signaling pathway downstream of EGFR may be involved. In summary, our findings have improved the understanding of the role of miR-7 in promoting chemosensitivity of cancer cells to PTX, and has enhanced existing knowledge on miRNAs in modulating chemotherapeutic efficacy. The identification of miR-7 as a potential sensitizer in PTX therapy provides a fundamental basis for new approaches in the development of novel and PTX therapeutic strategies.

## Figures and Tables

**Table I:** Primers used to detect the expression of U6, miR-7, GAPDH and EGFR by quantitative polymerase chain reaction.

Primer	Sequence (5′-3′)
U6	F: TCAGTTTGCTGTTCTGGGTG R: CGGTTGGCTGGAAAGGAG
miR-7	F: GGAAAGGCTCATTCGGACTA R: ACGACGCCACCAATCACT
GAPDH	F: TGCACCACCAACTGCTTAGC R: GGCATGGACTGTGGTCATGAG
EGFR	F: GCG TTCGGCACG GTGTATAA R: GGCTTTCGGAGATGTTGCTTC

miR-7, microRNA-7; EGFR, epidermal growth factor receptor.
